# Cardiovascular health and longevity: where are we?

**DOI:** 10.3389/fcvm.2026.1921582

**Published:** 2026-10-22

**Authors:** Amith Seri, Bing Yue, Muzamil Khawaja, W. H. Wilson Tang, Yochai Birnbaum, Carl J. Lavie, Salim S. Virani, Markus Strauss, Chayakrit Krittanawong

**Affiliations:** 1 Department of Hospital Medicine, West Virginia University/Princeton Community Hospital, Princeton, WV, United States; 2 PeaceHealth Southwest Medical Center, Vancouver, WA, United States; 3 Division of Cardiology, Emory University School of Medicine, Atlanta, GA, United States; 4 Department of Cardiovascular Medicine, Heart Vascular and Thoracic Institute, Cleveland, Clinic, Cleveland, OH, United States; 5 Section of Cardiology, Baylor College of Medicine, Houston, TX, United States; 6 John Ochsner Heart and Vascular Institute, Ochsner Clinical School, The University of Queensland School of Medicine, New Orleans, LA, United States; 7 Section of Cardiology, Department of Medicine, Pakistan & Department of Population Health, Aga Khan University Medical College, Karachi, Aga Khan University Medical College, Nairobi, Kenya; 8 Department of Cardiology I- Coronary and Peripheral Vascular Disease, Heart Failure Medicine, University Hospital Muenster, Cardiol, Muenster, Germany; 9 HumanX, Delaware, DE, United States

**Keywords:** cardiovascular aging, cardiovascular health, cardiovascular health and longevity, lifespan, longevity

## Abstract

Cardiovascular health is a key factor in human longevity, affecting not just lifespan but also the quality of aging. We synthesize evidence across four risk factor domains: genetic and epigenetic factors (APOE, FOXO3, telomere biology, epigenetic clocks); metabolic and vascular factors (hyperglycemia, dyslipidemia, obesity, arterial stiffness); lifestyle determinants (diet, physical activity, smoking, sleep, gut microbiome); and environmental exposure (air and noise pollution, climate stressors, ionizing radiation). These factors influence various biological processes such as oxidative stress, chronic low grade inflammation (inflammaging), mitochondrial dysfunction, metabolic disturbances, and changes in telomere length and epigenetics that collectively damage the endothelium, stiffen arteries, promote atherosclerosis, and contribute to overall cardiovascular decline with age. Understanding these processes helps explain why cardiovascular aging is such a strong predictor of mortality. We further summarize the principal intervention strategies with evidence for improving cardiovascular health and longevity, such as adherence to the American Heart Association's Life's Essential 8 construct, heart-healthy dietary patterns(Mediterranean, DASH), smoking cessation, sleep optimization, and reduction of environmental exposures. This narrative review distinguishes evidence derived from model organisms, human observational cohorts, and human interventional/genetic studies, since these evidence tiers differ substantially in their support for lifespan or longevity extending claims specifically, as opposed to cardiovascular risk reduction more broadly.

## Introduction

Longevity, or lifespan, is closely linked to aging processes, especially those impacting cardiovascular health. As the global population ages, cardiovascular disease (CVD) incidence increases, underscoring the importance of understanding how the cardiovascular system ages to promote healthier, longer lives. Cardiovascular aging involves various physiological changes that elevate the risk of heart failure and vascular issues, particularly in individuals over 80 years old (1). Recent research has provided insight into the complex connections between longevity and cardiovascular health (CVH) (2).

The American Heart Association defines CVH using the Life's Essential 8 (LE8) framework, which updates the previous Life's Simple 7. LE8 includes eight metrics: healthy diet, physical activity, avoiding nicotine, sleep health, body mass index, blood lipids, blood glucose, and blood pressure. Each metric is scored from 0 to 100, and the overall CVH score is calculated as the unweighted average of these eight components (3). In a nationally representative sample of 23,003 US adults followed for mortality outcomes, those with high cardiovascular health (LE8 score ≥80) had a life expectancy at age 50 of 36.2 years. In contrast, individuals with low CVH (LE8 score <50) had a life expectancy of only 27.3 years, a difference of 8.9 years. About 43% of this increase is linked to lower cardiovascular mortality (2). This association persists into extreme old age. In a combined analysis of the China Kadoorie Biobank and the China Hainan Centenarian Cohort including 31,473 adults aged 30 to 116 years, higher CVH was associated with lower all-cause mortality at every life stage, including a 54.8% lower risk among centenarians, with physical activity and body mass emerging as the most influential components in this group (4). Throughout this review, LE8 is used as a key reference because its components directly relate to the determinants discussed in the upcoming sections. Table 1 summarizes key studies linking each LE8 component to cardiovascular and longevity outcomes, several of which are discussed in more detail in the later sections.

**Table 1:** Key studies linking life's essential 8 (LE8) to cardiovascular and longevity outcomes.

Study	Population/Design	LE8 Exposure	Key Finding
Lloyd-Jones DM, et al. “Life's Essential 8: Updating and Enhancing the American Heart Association's Construct of Cardiovascular Health: A Presidential Advisory From the American Heart Association.” (3)	AHA Presidential Advisory; construct definition and validation cohorts	LE8 score 0–100 (8 metrics)	Established LE8 as the updated CVH construct; higher scores associated with lower CVD incidence and mortality across validation cohorts
Ma H, et al. “Cardiovascular Health and Life Expectancy Among Adults in the United States.” (2)	NHANES, US adults (nationally representative), *n* = 23,003	LE8 score quintiles	Higher LE8 score associated with greater life expectancy at age 50 — 36.2 years (high CVH) vs. 27.3 years (low CVH), an 8.9-year gain, ∼43% attributable to reduced cardiovascular mortality
Tessier AJ, et al. “Optimal Dietary Patterns for Healthy Aging.” (89)	Large US prospective cohorts (NHS + HPFS), ∼30-year follow-up, *n* = 105,015	Diet component of LE8 (AHEI, aMED, DASH, MIND, and four additional pattern-based scores)	Greater adherence to healthy dietary patterns associated with higher odds of healthy aging; AHEI showed the strongest association among all scores tested
Watts EL, et al. “Association of Leisure Time Physical Activity Types and Risks of All-Cause, Cardiovascular, and Cancer Mortality Among Older Adults.” (110)	Older US adults, prospective cohort	Physical-activity component of LE8	Moderate-to-vigorous activity associated with reduced all-cause and cardiovascular mortality; benefit plateaus at higher intensities
Li H, et al. “Association of Healthy Sleep Patterns With Risk of Mortality and Life Expectancy at Age of 30 Years: A Population-Based Cohort Study.” (129)	US population-based cohort	Sleep-health component of LE8	Healthy sleep pattern associated with ∼4.7 additional years of life in men and ∼2.4 years in women vs. poor sleep pattern
Mons U, et al. “Impact of Smoking and Smoking Cessation on Cardiovascular Events and Mortality Among Older Adults: Meta-Analysis of Individual Participant Data From Prospective Cohort Studies of the CHANCES Consortium.” (118)	Pooled prospective cohorts, older adults (CHANCES consortium)	Nicotine-exposure component of LE8	Smoking cessation associated with markedly reduced recurrent cardiovascular events and mortality vs. continued smoking
Rapsomaniki E, et al. “Blood Pressure and Incidence of Twelve Cardiovascular Diseases: Lifetime Risks, Healthy Life-Years Lost, and Age-Specific Associations in 1.25 Million People.” (167)	Population-based cohort (CALIBER), 1.25 million people, UK	Blood pressure component of LE8	Elevated blood pressure associated with earlier onset of cardiovascular disease and substantial loss of healthy life-years across the lifespan, with associations present even at BP levels currently considered normal
Prospective Studies Collaboration. “Blood Cholesterol and Vascular Mortality by Age, Sex, and Blood Pressure: A Meta-Analysis of Individual Data From 61 Prospective Studies With 55,000 Vascular Deaths.” (168)	Meta-analysis, ∼900,000 adults, 61 prospective studies	Blood lipids component of LE8	Higher total cholesterol associated with increased ischaemic heart disease mortality across middle and old age and at all blood pressure levels
Emerging Risk Factors Collaboration (Seshasai SR, et al.). “Diabetes Mellitus, Fasting Glucose, and Risk of Cause-Specific Death.” (169)	Meta-analysis, 820,900 people, 12.3 million person-years	Blood glucose component of LE8	Diabetes and elevated fasting glucose independently associated with increased vascular and all-cause mortality risk, beyond established risk factors
Chen S, et al. “Life's Essential 8 and Longevity: The Sustained Impact of Cardiovascular Health on Mortality from Middle Age to Centenarians” (4)	China Kadoorie Biobank (Hainan) + China Hainan Centenarian Cohort, *n* = 31,473; aged 30–116	LE8 score (low, moderate, high)	Higher LE8 score associated with lower all-cause mortality at every life stage, including 54.8% lower risk among centenarians; physical activity and body mass were the most influential components at extreme ages

This review expands on two recent comprehensive reviews. Pietri and Stefanadis connected cardiovascular aging to longevity by examining genetic, dietary, and environmental factors in long-lived populations. However, their work predates the AHA's Life's Essential 8 framework (2022) and does not include aspects such as sleep, noise pollution, the gut microbiome-TMAO pathway, or distinguish between the evidence for cardiovascular aging and that for longevity (5). Ministrini et al. reviewed molecular indicators of cardiovascular aging and how they can be influenced pharmacologically, focusing on clinical events rather than lifespan, while not considering genetic longevity factors, lifestyle choices, or environmental influences (6). This review uniquely integrates these determinants within a single LE8-based framework, clearly distinguishing the evidence supporting cardiovascular aging from the often less conclusive data on longevity. It also offers a comparative overview of heart-healthy dietary patterns. As a narrative rather than a systematic review, literature was gathered through PubMed searches from inception to August 2026, using keywords such as ‘cardiovascular health’, ‘longevity’, ‘cardiovascular aging’, and mechanism-specific terms like oxidative stress, telomeres, aging genes, and clonal hematopoiesis. Emphasis was placed on large cohort studies and meta-analyses for outcome data, as well as foundational or model-organism research for mechanisms, with levels of evidence indicated throughout.

This review carefully distinguishes several related terms rather than using them interchangeably. ‘Lifespan’ and ‘longevity’ both refer to the length of life. ‘Life expectancy’ is a specific actuarial or cohort-based estimate of remaining years at a certain age, as shown in life tables or survival analyses. ’Survival' refers to the precise endpoint measured in each study, such as all-cause, cardiovascular, or disease-specific survival, and is mentioned as such whenever discussed. ‘Healthy aging’ describes surviving to an older age while maintaining functional independence and avoiding major chronic diseases. ‘Cardiovascular health’ (CVH) specifically pertains to the American Heart Association's Life's Essential 8 framework. Findings from model organisms or preclinical research are clearly identified instead of being generalized into lifespan extension claims for humans, and observational data are presented as associations rather than proven causal effects.

The review has three main sections: factors influencing cardiovascular health, underlying mechanisms, and evidence-based interventions for improvement. Figure 1 provides a schematic overview of these determinants and their interactions. Figure 2 details the mechanisms by which the principal genetic and epigenetic factors influence cardiovascular health.

**Figure 1:**
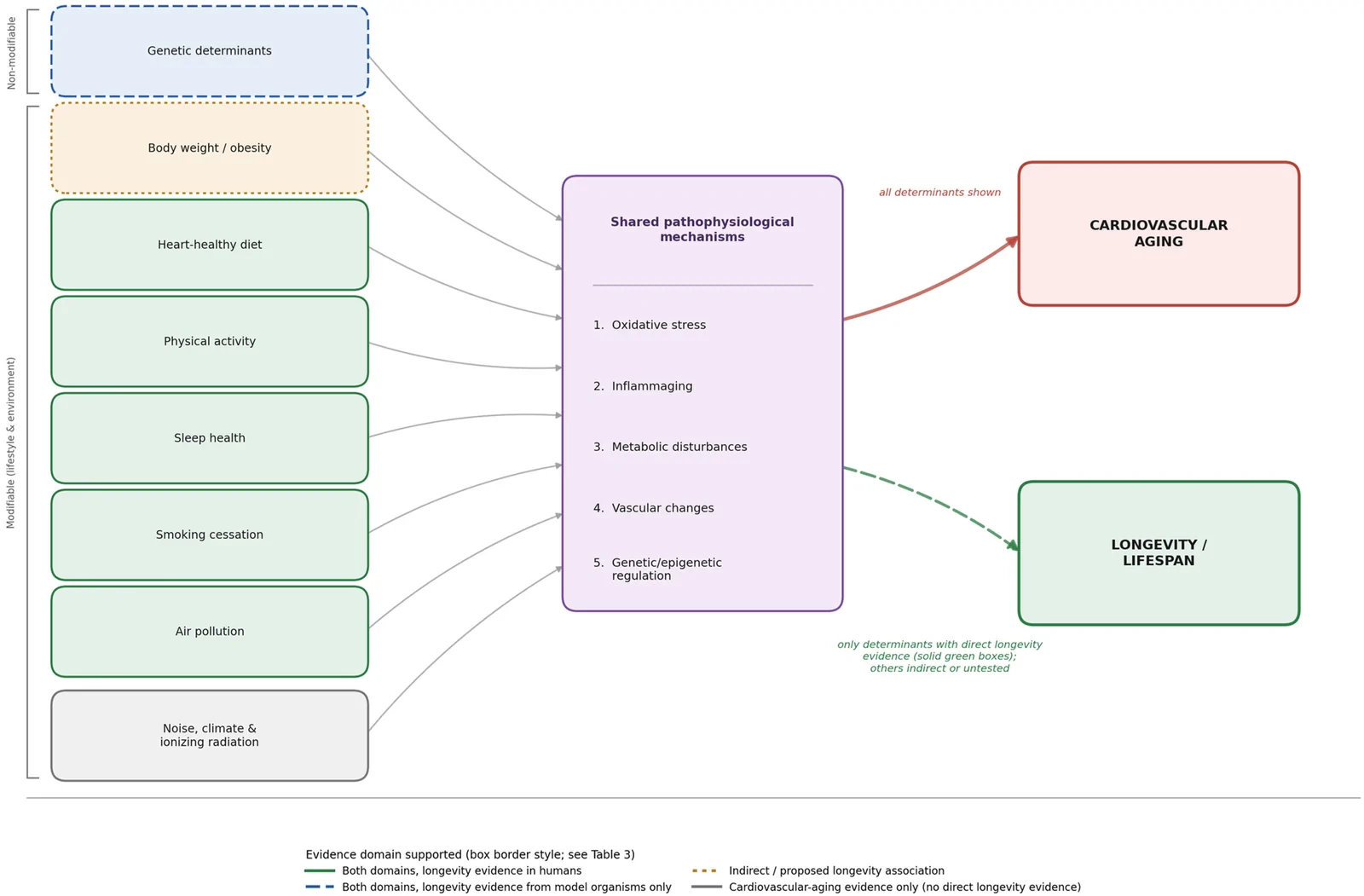
Schematic overview of the determinants of cardiovascular health and their evidence domains. Determinants are grouped as non-modifiable (genetic) or modifiable (lifestyle and environmental) and converge on shared pathophysiological mechanisms. Box border style indicates the evidence domain supported for each determinant, corresponding to Table 2: solid green, independent human evidence for both cardiovascular aging and a longevity endpoint; dashed blue, both domains but with lifespan-extension evidence from model organisms only; dotted amber, indirect or proposed longevity association; solid gray, cardiovascular-aging evidence only, with no direct longevity evidence. The solid arrow indicates that all determinants shown have evidence for cardiovascular aging; the dashed arrow indicates that only determinants with direct longevity evidence support the longevity/lifespan endpoint.

**Figure 2:**
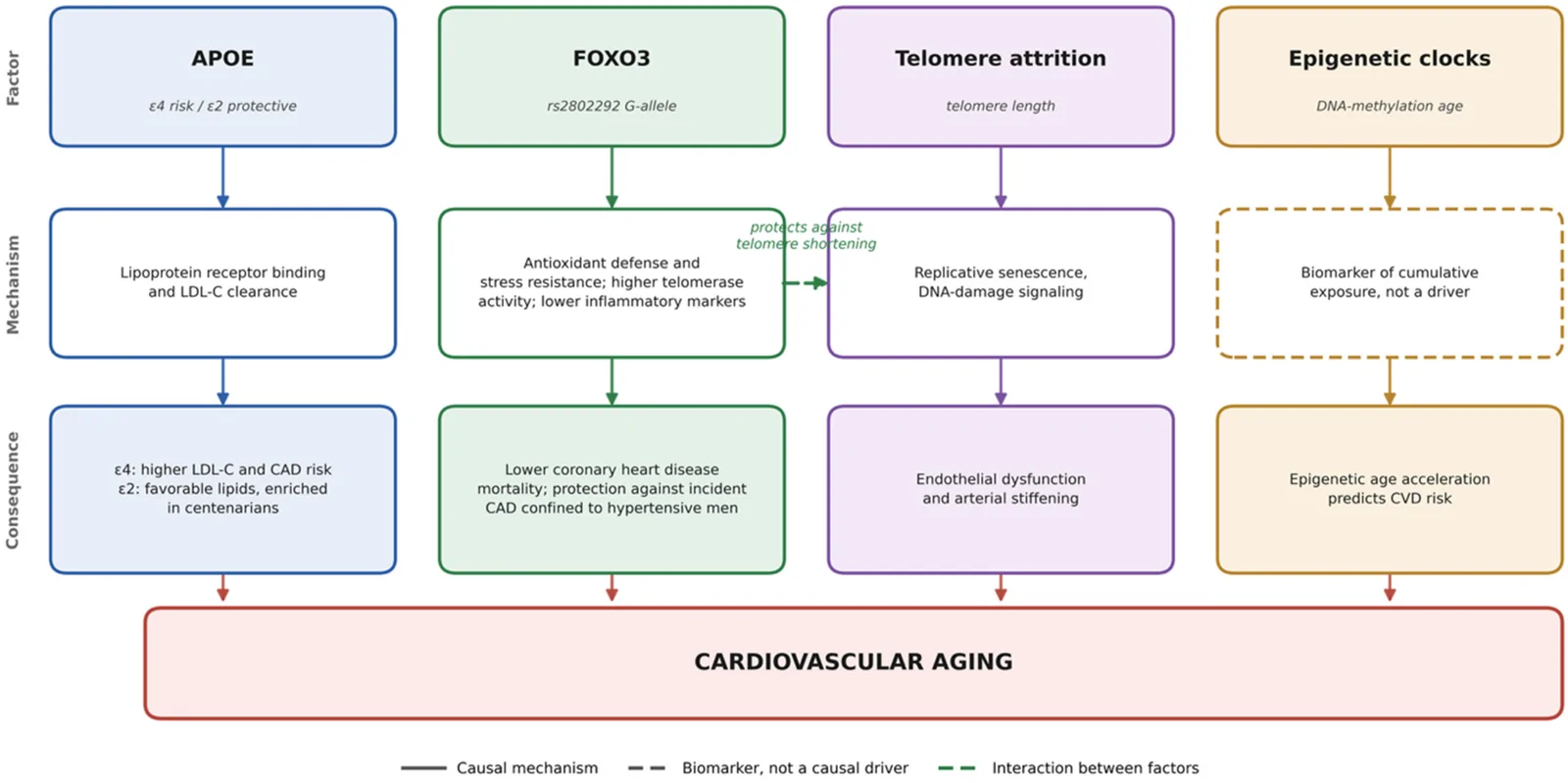
Mechanisms linking genetic and epigenetic factors to cardiovascular health. Each column traces one factor from the gene or marker, through its principal mechanism, to its cardiovascular consequence, with all four converging on cardiovascular aging. Solid borders indicate causal mechanisms; the dashed border for epigenetic clocks reflects that DNA-methylation age is an integrative biomarker rather than a causal driver. The green dashed arrow shows a mechanistic interaction between factors: carriers of the FOXO3 longevity variant rs2802292 exhibit higher telomerase activity and are protected against telomere attrition. LDL-C, low-density lipoprotein cholesterol; CAD, coronary artery disease; CVD, cardiovascular disease.

### Aging genes

This review begins with genetic pathways that control cellular and organismal aging, many of which directly influence cardiovascular outcomes, as discussed here. Aging affects physiological functions and is characterized by the accumulation of damage in molecules, cells, and tissues over time. This process often reduces an organism's ability to maintain homeostasis under stress and increases the risk of many diseases (cancer, cardiovascular and neurodegenerative disorders) and early death (7). Understanding what influences aging is difficult because of the process's complexity and because there is significant variability among individuals and even between tissues within the same body. At the cellular level, cell senescence is the most noticeable change in aging tissue, caused by exposure to both internal and external aging factors. It is marked by a gradual buildup of DNA damage and epigenetic modifications that alter DNA structure, disrupt gene expression, and impair cell function (8). Mutations in specific genes can enhance stress resistance and slow damage accumulation. The adaptor protein p66Shc is crucial in promoting cellular senescence by regulating mitochondrial reactive oxygen species (ROS) levels, which affect cell death (9). Since this ROS production also directly triggers endothelial dysfunction and early atherosclerotic plaque formation, p66Shc connects cellular senescence specifically to vascular aging, rather than aging overall. Genetic factors greatly shape aging, with specific gene combinations affecting lifespan; even minor changes can cause significant differences in lifespan, as observed in human progeroid syndromes (10). Twin studies and genome association research in centenarians emphasize the importance of genetic variability in aging and related diseases (7). Mutations in nuclear and mitochondrial DNA, coupled with reduced repair capacity, lead to a decline in somatic stem cell function (11). Notable findings from model organisms like Caenorhabditis elegans demonstrate that longevity can be extended through conserved metabolic and stress response pathways. Mutations affecting mitochondrial function, including clk-1, which regulates ubiquinone biosynthesis and metabolic timing, and the iron sulfur protein 1 (isp-1), which partially reduced mitochondrial electron transport, lower oxidative stress and slow metabolic rates, thereby promoting lifespan extension. In parallel, reduced insulin/IGF-I signaling pathway through daf-2 extends lifespan by relieving inhibitory phosphorylation of the transcription factor DAF-16, enabling its nuclear translocation and activation of gene programs involved in stress resistance, detoxification, metabolic remodeling and cellular homeostasis (12, 13). The mammalian target of rapamycin (mTOR) is a highly conserved serine/threonine kinase that functions through two distinct multiprotein complexes to integrate nutrient, energy, and hormonal signals, thereby regulating cell growth and metabolism. Inhibiting mTOR signaling has been shown to extend lifespan in various model organisms in part through induction of autophagy, highlighting its connection to insulin and nutrient sensing pathways, though this lifespan benefit has not been demonstrated in humans (14). With age, persistent activation of mTOR complex 1 (mTORC1), the nutrient-sensitive component that promotes anabolic metabolism, suppresses autophagy and cellular repair, contributing to metabolic dysfunction and accumulation of cellular damage. This pathway is particularly relevant to cardiovascular aging, as mTORC1 hyperactivation contributes to vascular smooth muscle cell aging and arterial stiffening; whether attenuating mTOR activity extends lifespan or improves vascular aging in humans remains to be established, though this relationship is supported in preclinical models (15). Sirtuins are enzymes that depend on NAD⁺ and control important cell functions such as metabolism, DNA repair, inflammation and stress response. They are crucial in managing basic aging processes. Their activity declines with age, largely due to falling intracellular NAD + levels, which impairs mitochondrial functions and accelerates vascular and metabolic aging (16). Strategies to restore NAD⁺ availability are being actively investigated, though clinical translation remains at an early stage (17). Since sirtuins also support endothelial nitric oxide signaling and help in blood vessel repair, the decline in their activity with age particularly accelerates vascular aging, beyond just metabolic aging. These pathways, which include redox and mitochondrial regulation (p66Shc), nutrient sensing and growth signaling (DAF-16, mTOR), and NAD⁺-dependent stress resistance (sirtuins), all converge on the same processes that drive cardiovascular aging, rather than being separate aging mechanisms unrelated to the cardiovascular system.

### Longevity genes

Longevity genes are associated with extended lifespan and enhanced resistance to age-related diseases. Research indicates that genetic factors contribute to longevity, with heritability estimates ranging from 10% to 30% based on twin and pedigree studies (18). Most human genetic variation originates from single-nucleotide polymorphisms (SNPs), although other types of genetic changes, such as insertions, also occur (19). Early candidate gene studies focused on specific variants, notably identifying apolipoprotein E (APOE) as a crucial longevity locus. Schachter et al. reported that French centenarians exhibited depletion of ApoE *ε*4 and enrichment of ApoE *ε*2, a pattern they attributed to long-term survival selection driven primarily by cardiovascular risk. ApoE *ε*4 is associated with elevated LDL-cholesterol and increased risk of both coronary artery disease and Alzheimer's disease. Whereas ApoE *ε*2 is generally linked to a more favorable lipid profile and reduced coronary risk for most carriers (20). Additionally, forkhead box protein O (FOXO)3 has emerged as another frequently associated locus, initially reported by Willcox et al. (21). FOXO3 target genes control antioxidant defense and stress resistance in vascular tissue, providing this longevity locus a direct means to protect against vascular aging, not merely overall longevity. The cardiovascular contribution to this association has since been quantified. In a 17-year prospective study involving older American men of Japanese descent, with validation in white and black individuals, carriage of the longevity associated G allele of rs2802292 was associated with a 10% reduction in all-cause mortality. This reduction was mainly due to a 26% lower risk of death from coronary heart disease; no other causes significantly influenced the survival benefit (22). A companion analysis verified that protection against coronary artery disease mortality was consistent across all three populations. It estimated that the non-protective TT genotype contributed to 15%, 9%, and 3% of coronary mortality risk in Japanese, white, and Black Americans, respectively. This contribution is similar to the impact of hypertension observed in the Japanese group. Additionally, carriers of the G-allele exhibited lower plasma levels of tumor necrosis factor-α, suggesting that inflammation might play a mediating role (23). This protection appears conditional rather than uniform. In middle-aged men tracked from midlife in the Kuakini Honolulu Heart Program, the G allele did not independently lower the risk of coronary artery disease. Instead, it significantly interacted with hypertension and provided protection only in hypertensive men, indicating that the variant helps buffer cardiovascular risk under physiological stress rather than reducing baseline risk (24). At the cellular level, individuals aged 55 and above who carry the G allele exhibit protection against telomere shortening in peripheral blood mononuclear cells. This is accompanied by increased telomerase activity and sex-specific variations in inflammatory markers (25). Consequently, FOXO3 might impact cardiovascular aging through multiple partly independent pathways, including redox defense, telomere preservation, and inflammatory regulation. Beyond individual loci, researchers expanded their focus to pathway-based analyses, recognizing that variants within the same biological pathways can exert similar effects. For instance, Deelen et al. identified associations with SNPs in the insulin/IGF-1 signaling (IIS) and telomere maintenance pathways, while Debrabant et al. reported only minimal associations in DNA repair pathways (26, 27). More recently, Dato et al. uncovered significant SNP-SNP interactions involving Tumor Protein p53 (TP53) and Growth Hormone Secretagogue Receptor (GHSR) across IIS and antioxidant pathways (28). Genotyping arrays made it possible to scan the entire genome at once, shifting longevity research from studying a few suspected genes to using unbiased methods like linkage analysis and genome-wide association studies (GWAS) to discover new genetic signals. While linkage studies aimed to identify key loci in long-lived families, they have seen limited success, primarily due to small sample sizes (29). In contrast, GWAS have identified several variants associated with longevity, although the APOE locus remains the only signal consistently replicated across different populations (30, 31). Overall, the genetic basis of human longevity centers around a few key pathways-lipid management (APOE), redox and stress resistance (variants in FOXO3 and related antioxidant pathways), and nutrient sensing signaling (interactions involving IIS). These pathways are the same ones involved in the mechanisms of cardiovascular aging, rather than forming a distinct set of longevity genes independent of cardiovascular health.

### Pathophysiological links between cardiovascular aging and longevity

The genetic factors mentioned earlier influence both cardiovascular aging and longevity via common biological mechanisms. These processes are interconnected, as they impact both vascular health and lifespan through overlapping pathways (5). Five such mechanisms recur throughout this literature—oxidative stress, chronic low-grade inflammation, metabolic disturbances, vascular changes, and genetic–epigenetic regulation. Table 2 summarizes the evidence tier, consistency and clinical maturity for each mechanistic factor discussed below.

**Table 2:** Evidence table: mechanistic factors linking cardiovascular aging and longevity.

Factor	Evidence domain supported	Primary outcome measured	Evidence tier	Directional consistency	Key limitation	Clinical maturity
Aging genes/conserved longevity pathways (mTOR, sirtuins, insulin/IGF-1 signaling)	Both (longevity: model organism only)	Lifespan (C. elegans, Drosophila, mice); vascular smooth muscle aging and arterial stiffening (mechanistic, human)	Model organism (daf-2/DAF-16, mTOR, sirtuin animal studies) (12–16); human evidence limited to downstream mechanistic pathways	Consistent lifespan extension across multiple model organisms; human translational effect not demonstrated	mTOR inhibitors are used in humans for other conditions (e.g., transplant medicine, certain cancers, and are in trials as anti-epileptic agents), but no human trial has tested mTOR inhibition or sirtuin activation specifically for lifespan extension; that evidence remains limited to model organisms	Research-stage for longevity specifically; drugs targeting these pathways carry known trade-offs (e.g., immune suppression) and are not used clinically for this purpose
Oxidative stress/mitohormesis	Both (longevity: model organism only)	Cardiomyopathy resistance, lifespan (model organisms); antioxidant capacity (humans)	Model organism (AC-5 KO mice (36); developmental ROS/lifespan in C. elegans (37); mitohormesis (39)); human observational (centenarian antioxidant study) (38)	Consistent direction across model and human observational data	No human RCT has tested whether modulating this pathway extends lifespan; direct lifespan-extension evidence is from model organisms, supported only by human genetic-association data (IGF-1 signaling variants linked to human longevity), not intervention studies	Research-stage; not yet a basis for clinical antioxidant therapy recommendations
Inflammaging (NF-*κ*B, IL-6, IL-1)	Both (longevity: model organism only)	CVD incidence, mortality (humans); survival (Drosophila)	Human observational (42–44); human Mendelian randomization (45); human RCT (48); model organism (Drosophila (46); mice (47))	Consistent association; canakinumab RCT confirms causal role in CV events	Canakinumab reduces cardiovascular events, but was not shown to extend overall lifespan in the trial that tested it; survival extension by NF- κB inhibition is reported only in Drosophila	Anti-inflammatory therapy is used clinically for cardiovascular risk reduction, but not prescribed specifically for longevity
Metabolic dysregulation	Both (longevity: model organism only)	Lifespan (C. elegans); insulin sensitivity, LDL-C (humans)	Model organism (49, 50, 93); human observational (51); human RCT (PROSPER) (56)	Consistent for CV risk; inconsistent for lifespan extension in humans	Calorie restriction's lifespan effect is mainly evidenced in model organisms; statin evidence in humans addresses a different pathway and has not shown a lifespan benefit	Statins are standard clinical therapy for cardiovascular risk; calorie restriction is not a standardized clinical recommendation for longevity
Vascular changes	Both (human longevity)	Pulse wave velocity, CV mortality, survival beyond 90	Human observational (65, 66); human genetic association (63, 64); familial cohort (67)	Consistent across genetic and observational studies	Identified genetic effect sizes are small, and no treatment directly targeting arterial stiffness has been proven to extend lifespan	Arterial stiffness (PWV) is used as a research and risk-stratification marker but is not yet a standard treatment target
Genetic–epigenetic regulation	Indirect/proposed	Epigenetic age, telomere length, incident CVD/mortality	Human observational (73–75) (79, 82); model organism (telomerase mechanism) (78)	Consistent association between accelerated epigenetic age/CHIP and CV risk	Testing methods and interpretation of epigenetic clocks are not standardized across laboratories	Not yet used in routine clinical care; remains a research tool
Obesity/obesity paradox	Indirect/proposed	All-cause and CV mortality by BMI/adiposity	Human observational, heterogeneous (84–88)	Inconsistent, attributable to measurement bias and confounding	Most large studies rely on BMI alone, which cannot distinguish fat distribution, visceral fat, or muscle mass, and results are confounded by smoking status and reverse causation. Whether the paradox reflects a genuine association or a measurement artifact remains actively debated.	Not yet actionable as a specific clinical strategy; better body-composition-based study designs are needed
Heart-healthy diet	Both (human longevity)	All-cause mortality, healthy aging, CV mortality	Human observational, large cohorts (89) (105, 106); human RCT (microbiome/TMAO) (104)	Highly consistent	Most evidence is observational; the available RCT evidence addresses a narrower mechanistic question (gut microbiome/TMAO) rather than long-term mortality directly	Among the most mature and actionable interventions in this review, already reflected in standard dietary guidelines
Physical activity	Both (human longevity)	All-cause/CV mortality; telomere length	Human observational (110, 112, 113); human association for telomere length (115, 116)	Consistent for mortality; telomere association weaker	The link between physical activity and cellular aging markers like telomere length is correlational, not based on intervention trials	Well established and actionable for cardiovascular risk reduction; claims about cellular/longevity effects remain preliminary
Smoking cessation	Both (human longevity)	Recurrent CV events, mortality, life expectancy	Human observational, large cohorts (118) (120, 121)	Highly consistent	Former smokers continue to carry somewhat higher cardiovascular risk than people who never smoked	Well established and actionable; a standard clinical recommendation
Sleep duration/quality	Both (human longevity)	CHD risk, CVD mortality, life expectancy	Human observational, meta-analyses (123, 125, 128, 129)	Consistent U-shaped risk curve	Few randomized controlled trials have directly tested whether improving sleep extends lifespan	Recommending 7–9 h of sleep is reasonable and widely used in clinical guidance, though not backed by outcome-based RCTs
Air pollution	Both (human longevity)	Life expectancy, CV mortality, subclinical atherosclerosis	Human observational, large cohorts (132, 136–138)	Highly consistent	Life expectancy estimates rely on indirect calculations rather than direct trial data, though independent methods produce consistent results	Actionable mainly at the policy level (air quality regulation); limited options for individual-level mitigation
Noise pollution	CV-aging only	Hypertension, diabetes, atherosclerosis incidence	Human observational (141, 142)	Consistent but smaller effect sizes; no direct longevity data	No study has directly measured an effect of noise exposure on lifespan; current evidence covers intermediate risk factors only	Not yet actionable as a specific clinical target; relevant mainly for policy and environmental health guidance
Climate (heat/cold stress, extreme weather, climate-linked air quality)	CV-aging only	Acute CV events (MI, arrhythmia, heart failure exacerbation), CV mortality; metabolic disorder incidence	Human observational, epidemiological and natural-experiment studies (143–145, 147, 148); ecological (microplastics/pollution overlap)	Consistent for acute CV event risk during temperature extremes and extreme weather	No study has directly measured an effect on human lifespan; the link to biological aging is inferred from shared mechanisms, and effects fall unevenly on disadvantaged populations, complicating causal attribution	Not clinically actionable at the individual level; response requires population and policy-level strategies
Ionizing radiation — high dose	CV-aging only	CV mortality, atherosclerosis, cardiomyopathy	Human observational, atomic-bomb and radiotherapy cohorts (149)	Consistent, dose-dependent	Evidence comes from specific high-exposure groups and may not generalize to typical background exposure in the general population	Well established for high-exposure clinical and occupational settings; not relevant to general-population longevity guidance
Ionizing radiation — low dose/hormesis	Indirect/proposed	CV/other health risk at low dose	Mixed: model-organism/ecological (151, 152, 156, 157); human epi meta-analyses (153, 154)	Inconsistent/actively debated	Ecological studies cannot establish causality, and findings from model organisms and human epidemiology point in different directions	Unresolved; no clinical guidance can be derived from this evidence at present

Evidence domain supported: CV-aging only = evidence addresses cardiovascular aging or intermediate cardiovascular risk factors, with no direct longevity/lifespan endpoint. Both (human longevity) = independent human evidence exists for both cardiovascular aging and a longevity endpoint (survival, life expectancy, or healthy aging), each with its own supporting reference. Both (longevity: model organism only) = cardiovascular-aging evidence is human, but lifespan-extension evidence derives from model organisms and has not been demonstrated in humans. Indirect/proposed = a longevity association is proposed but is confounded, inconsistent, or inferred rather than independently established.

**Table 3:** Comparison of major heart-healthy dietary patterns.

Dietary Pattern	Key Components	Outcome
Mediterranean diet	High olive oil, vegetables, fruit, legumes, fish, whole grains; moderate wine; low red/processed meat	Reduced cardiovascular and all-cause mortality on long-term cohort follow-up (105) (106). In a randomized crossover feeding trial, a Mediterranean-style pattern with modest lean beef intake increased gut microbiota diversity and lowered plasma and urinary TMAO relative to an average American diet (104).
AHEI (Alternative Healthy Eating Index)	Composite score emphasizing vegetables, fruit, whole grains, nuts/legumes, unsaturated fat; penalizes red/processed meat, sugar-sweetened drinks, sodium	Of eight dietary pattern scores compared head-to-head in large US prospective cohorts — spanning conventional diet patterns as well as plant-based and biomarker-derived inflammatory/insulinemic indices — AHEI showed the strongest association with healthy aging, supporting its use as a benchmark measure of overall diet quality (89)
aMED (Alternative Mediterranean Index)	Adapted Mediterranean scoring calibrated to non-Mediterranean populations	A Mediterranean-style eating pattern predicts healthy aging even in non-Mediterranean, US-based populations, indicating the pattern's benefits are not confined to the region it originates from (89).
DASH (Dietary Approaches to Stop Hypertension)	High fruit, vegetables, low-fat dairy, whole grains; low sodium, saturated fat, sweets	Beyond its well-established blood-pressure-lowering effect, DASH adherence independently predicts healthy aging in long-term cohort follow-up, indicating benefits that extend past hypertension control alone (89)
MIND (Mediterranean-DASH Intervention for Neurodegenerative Delay)	Hybrid of Mediterranean and DASH with emphasis on berries and leafy greens	Although designed specifically to reduce neurodegenerative risk, MIND diet adherence also predicts broader healthy aging outcomes, suggesting shared dietary drivers underlie cognitive and physical aging trajectories (89)
Okinawan diet	Low-calorie-density, plant-based, sweet potato staple; traditional portion control (“Hara Hachi Bu”, eating to ∼80% fullness); mild sustained caloric restriction	Associated with regional longevity and healthy aging in the Okinawa Centenarian Study population, though evidence is largely observational/ecological rather than interventional (91)

*Oxidative stress* plays a key role in aging. An imbalance between reactive oxygen species (ROS) and antioxidant defenses causes mitochondrial DNA damage, disrupts cellular metabolism, and activates senescence or apoptotic pathways (32–34). Research shows that inhibiting the type 5 isoform of adenylyl cyclase (AC-5), an important part of *β*-adrenergic signaling, improves resistance to oxidative stress by increasing superoxide dismutase levels, which protects against stress-induced cardiomyopathy and extends lifespan by nearly 30% (35, 36). Long-lived individuals and populations tend to exhibit lower levels of oxidative stress and better antioxidant capacity, reflecting both innate stress-resilience mechanisms and modifiable influences, including early-life redox programming, metabolic efficiency, and long-term regulation of antioxidant defenses (37, 38). New evidence supports the idea of mitohormesis, where brief or mild ROS exposure triggers adaptive responses that boost stress resistance and promote longevity (39).

Oxidative stress and inflammation are closely connected in their mechanisms. Continuous oxidative stress activates the redox-sensitive transcription factor NF-*κ*B, which Chung et al. identify as the main pathway promoting the senescence-associated secretory phenotype, thereby maintaining chronic low-grade inflammation with age (40). This process, known as inflammaging, is a persistent, low-level inflammation related to aging that links cardiovascular deterioration to reduced lifespan (41). Elevated levels of proinflammatory mediators like interleukin (IL)-1, IL-6, tumor necrosis factor-α, and C-reactive protein are associated with increased cardiovascular disease and death, while anti-inflammatory cytokines such as IL-10 are linked to exceptional longevity (42–44). Mendelian randomization studies have also linked inflammaging biomarkers to retinal microvascular structure, cardiovascular disease, and lifespan, offering genetic evidence that supports these observational findings (45). NF-*κ*B inhibition was found to extend survival in some animal studies on Drosophila (46). Factors like visceral fat, a source of inflammatory cytokines and hormones, and age-related changes in gut microbiota that enhance intestinal permeability and immune activation contribute to this process (41). Inflammation also promotes atherosclerosis, making it a crucial driver of cardiovascular aging. Drugs like renin angiotensin-aldosterone system (RAAS) inhibitors, statins, and aspirin provide part of their cardiovascular benefits through anti-inflammatory effects, with some also extending lifespan in experimental models (47). More recently, targeted cytokine therapies, such as IL-1β blockade with canakinumab, have been shown to reduce cardiovascular events, highlighting inflammation as both a mechanistic link and a treatment target in cardiovascular aging (48). This trial did not assess lifespan and survival extension through inflammatory pathway inhibition has so far been demonstrated only in model organisms.

Inflammation and metabolic dysfunction reinforce each other, as visceral adiposity, previously mentioned as a source of inflammatory cytokines, is also a key driver of the metabolic disturbances discussed here. These metabolic disturbances, particularly hyperglycemia, insulin resistance, and dyslipidemia, significantly contribute to cardiovascular aging and a reduced lifespan. Hyperglycemia speeds up aging through oxidative stress and advanced glycation end products, while excessive insulin/IGF-1 signaling blocks longevity-promoting pathways like adenosine monophosphate-activated protein kinase (AMPK), FOXO, and sirtuins, and activates mTOR, a major driver of cellular aging (49, 50). On the other hand, maintaining insulin sensitivity, low insulin levels, and higher adiponectin are typical of very long-lived people (51). Dyslipidemia, largely due to visceral adiposity, fosters inflammation and atherogenic lipoprotein production (52). In experimental models, Sirtuin-1 gain of function improves hepatic insulin sensitivity and glucose tolerance and raises adiponectin levels, without demonstrated effects on body weight or composition (53). Impaired regulation of 3-hydroxy-3-methylglutaryl coenzyme A (HMG-CoA) reductase contributes to hypercholesterolemia in aging, a process mitigated by calorie restriction (54, 55). However, this metabolic effect should be distinguished from pharmacologic HMG-CoA reductase inhibition, as statin therapy in older adults has not consistently been associated with lifespan extension, including in large trials like PROSPER (56). Genetic influences are also critical: APOE4 increases LDL-C, CVD, and Alzheimer's risk, whereas APOE2/3 and favorable LDL profiles are enriched in centenarians, who also display larger LDL and high-density lipoprotein (HDL) particles linked with reduced metabolic syndrome and cardiovascular risk (57–59).

*Vascular changes*, such as arterial hypertension and arterial stiffness, contribute to cardiovascular aging and are closely interconnected, with evidence supporting a bidirectional relationship between the two (60). Hypertension alone affects an estimated 1.39 billion adults worldwide (61). Both are linked to subclinical inflammation and oxidative stress, supporting the concept of inflammaging (62). Genetic variants associated with lower blood pressure have been linked to longevity. A meta-analysis identified the rs2149954 locus on chromosome 5q33.3 as associated with survival beyond 90 years, with carriers of the minor T allele showing lower cardiovascular mortality, primarily through stroke protection. This allele had also been previously linked to lower midlife blood pressure (63). Similarly, the rs198389 variant in the promoter region of the B-type natriuretic peptide gene (NPPB) gene has been associated with reduced blood pressure, lower cardiovascular mortality, and greater longevity (64). Arterial stiffness, primarily measured by carotid–femoral pulse wave velocity (PWV), serves as a crucial marker of early vascular aging and is an independent predictor of cardiovascular events and mortality (65, 66). Familial longevity has been related to lower PWV in offspring, indicating a heritable aspect of vascular health (67). The IKARIA study demonstrated that adults over 50 years of age had significantly lower PWV than age matched individuals in general population, indicating slower arterial stiffening and suggesting decelerated vascular aging influenced by genetic, environmental, and lifestyle factors (68).

Integrating *Genetic and Epigenetic mechanisms* deepens our understanding of cardiovascular aging and lifespan. While genetic factors influence key aging pathways, they do not solely determine aging outcomes. Epigenetics involves reversible changes in gene regulation without changing the DNA sequence. This layer of control translates lifestyle choices and environmental exposures into modifications in gene expression, enabling behaviors to impact cardiovascular aging. Epigenetic processes such as DNA methylation, histone modifications, chromatin remodeling, and noncoding RNA regulation accumulate with age, altering transcriptional programs that affect vascular health, metabolic resilience, and inflammation (69, 70). These epigenetic marks are highly responsive to lifestyle factors like diet, physical activity, smoking, and sleep, meaning positive behaviors can partly counteract genetic risks and support healthier cardiovascular aging (71, 72). Dysfunctions in these pathways contribute to endothelial problems, mitochondrial decline, inflammaging, and metabolic issues, heightening cardiovascular disease risk (69). Epigenetic clocks developed by Hannum and Horvath assess biological age using predictable DNA-methylation patterns. These clocks provide a way to quantify the rate at which the body is aging at a molecular level. When the methylation based biological age exceeds the chronological age, it indicates accelerated aging and has been linked to increased cardiovascular risk and mortality. These findings suggest that epigenetic clocks could serve as valuable markers of vascular aging, although their application in everyday clinical settings is still being determined (73–75). Figure 2 summarizes how these genetic and epigenetic factors—APOE, FOXO3, telomere attrition, and epigenetic clocks—affect cardiovascular health. It also distinguishes those that function as causal mechanisms from DNA methylation age, which acts as an integrated biomarker of cumulative exposure rather than a direct driver disease. Telomeres, protective DNA protein structures at chromosome ends, shorten progressively with each cell division because DNA polymerases are unable to replicate the final portion of each chromosome (76). This shortening is markedly accelerated by oxidative stress and chronic inflammation, two central drivers of atherosclerosis (32). As telomeres erode to a critically short threshold, they activate DNA-damage signaling pathways, particularly p53-mediated responses, triggering cellular senescence or apoptosis (77). This loss of regenerative capacity affects endothelial cells, vascular smooth muscle cells, and cardiomyocytes, thereby promoting endothelial dysfunction, arterial stiffening, impaired myocardial repair, and ultimately accelerating vascular aging. In experimental models, loss of telomerase leads to cardiac dilatation and heart failure, while in human cohorts a promoter variant associated with greater telomerase activity has been linked to longer survival (78, 79). Telomerase composed of reverse transcriptase component (hTERT) and RNA component (hTERC) can partially restore telomere length and confer additional protective effects independent of telomere elongation (78–81). Inflammation-driven pathways are also implicated in vascular aging: expression of specific inflammasome gene modules stratifies older adults by rates of hypertension and arterial stiffness, and in individuals over 85 years is associated with all-cause mortality (82). Relatedly, clonal hematopoiesis of indeterminate potential (CHIP), marked by somatic mutations in hematopoietic stem cells, has been linked to inflammation, vascular dysfunction, and higher mortality. This connects somatic mutations with both cardiovascular aging and increased mortality risk (83). Genetics determine predisposition, yet epigenetics provides the molecular flexibility for modifiable behaviors to reduce inherited risk and support healthier aging. Understanding this interaction is crucial for developing preventative strategies and personalized medicine approaches to slow vascular aging and promote a longer, healthier life.

### Obesity paradox phenomenon in cardiovascular aging and longevity

The metabolic and vascular mechanisms mentioned earlier are further complicated in older adults by an unresolved pattern linking body weight to cardiovascular outcomes. This issue must be addressed before focusing on dietary and lifestyle interventions. The obesity paradox describes the counterintuitive finding that overweight or obese patients with cardiovascular disease sometimes experience better short- to mid-term survival than normal-weight patients (84). Systematic reviews focusing on older adults show mixed results but often highlight a survival benefit for overweight or obese seniors, especially in studies involving patients with comorbidities or acute illnesses. Many of these reviews also note that the paradox is more frequently observed in studies examining short-term mortality or populations with specific medical conditions than in long-term, general population studies (85). A key methodological issue is that most large epidemiological studies rely solely on BMI as a measure of adiposity, which cannot differentiate fat distribution, visceral fat, or the important role of lean muscle mass. Another significant source of bias is confounding due to smoking, as smokers often have lower body weight but much higher baseline mortality. When the normal-weight group includes more current or former smokers, survival rates in higher-BMI categories can appear artificially improved. This can lead to misclassification of risk in older adults who have sarcopenia or central fat buildup (86). Obesity and aging share numerous biological pathways, including mitochondrial dysfunction, chronic inflammation, oxidative stress, impaired autophagy, genomic instability, and cellular senescence, which drive cardiac remodeling and accelerate vascular aging. At the organ level, obesity causes myocardial hypertrophy, fibrosis, and diastolic dysfunction, resembling age-related pathology. Meanwhile, the vasculature exhibits premature aging features, including endothelial dysfunction, arterial stiffening, and microvascular rarefaction, all of which increase long-term cardiovascular risk (87). Analyses using refined measures of visceral adiposity and adipose dysfunction show non-linear relationships with mortality, such as the J-shaped pattern seen with the Visceral Adiposity Index. This highlights that fat distribution and quality are prognostically more important in older adults than BMI alone (88). Resolving the obesity paradox requires accurate, long-term research that considers body composition, fat distribution, age, frailty, and mechanistic biomarkers of aging. Much of the paradox stems from measurement limitations and bias, while evidence links central adiposity to faster cardiovascular aging and increased long-term risk. These findings emphasize the need for personalized clinical strategies and targeted research instead of relying solely on BMI.

### Heart-healthy diet

Having outlined the genetic determinants and biological mechanisms of cardiovascular aging, this review now turns to evidence-based interventions, beginning with diet, the most extensively studied lifestyle factor in this context. Dietary intake influences cardiovascular aging not only through macronutrient composition but also through overall dietary pattern, food processing, portion size, and circadian timing of intake. Long-term adherence to healthy dietary patterns is increasingly seen as a crucial factor for healthy aging. In large prospective studies spanning 30 years, greater adherence to diets like the Alternative Healthy Eating Index (AHEI), Alternative Mediterranean Index (aMED), Dietary Approaches to Stop Hypertension (DASH), and the Mediterranean-DASH Intervention for Neurodegenerative Delay (MIND) was consistently linked to higher chances of healthy aging—defined as surviving to old age without major chronic illnesses and maintaining functional independence. Among these and four other pattern based scores tested in the same cohort (the healthy plant-based diet index [hPDI], planetary health diet index [PHDI], and reversed empirical dietary index for hyperinsulinemia and inflammatory pattern [rEDIH, rEDIP]), AHEI demonstrated the strongest link to healthy aging, highlighting its usefulness as a benchmark for overall diet quality (89). Table 3 summarizes the composition and reported cardiovascular and longevity outcomes for each of the dietary patterns discussed in this section. These diets generally emphasize high consumption of fruits, vegetables, whole grains, nuts, legumes, and unsaturated fats, while limiting red and processed meats, trans fats, sodium, and sugar-sweetened drinks. Conversely, higher intake of ultra-processed foods was independently connected to significantly reduced odds of healthy aging (89). Maintaining a stable, lean body weight throughout adulthood is consistently associated with lower all-cause and cardiovascular mortality, reduced cardiometabolic disease risk, and a higher likelihood of healthy aging. Even modest, sustained weight gain in early to mid-adulthood was associated with increased risk of diabetes, hypertension, cardiovascular disease, and premature mortality (90). Traditional longevity-associated dietary patterns, such as the Okinawan diet, incorporate portion control practices, including eating until 80% full, known as Hara Hachi Bu, which mimics lifelong mild caloric restriction without malnutrition (91). Calorie restriction without malnutrition extends lifespan in non-human primates and other model organisms and slows the development of age-related disease (92, 93). In humans, sustained calorie restriction improves cardiometabolic risk factors, though a direct lifespan-extending effect has not been demonstrated. These effects are thought to be mediated through coordinated regulation of conserved nutrient-sensing pathways, including decreased IGF-1 signaling, lowered mTOR activity, activated AMPK and sirtuin-1 (SIRT1), enhanced NAD⁺-dependent pathways and autophagy, and reduced chronic inflammation and oxidative stress (94, 95). Circadian rhythms are central regulators of aging, coordinating metabolic homeostasis, cellular repair and stress response pathways. Circadian rhythm aligned feeding–fasting cycles, particularly time-restricted feeding, enhance clock-controlled autophagy and metabolic efficiency, whereas circadian disruption accelerates cardiometabolic dysfunction, inflammation, and functional decline, underscoring circadian integrity as a conserved determinant of healthy aging (96).

Aging significantly affects the composition and function of the gut microbiome, a flexible ecosystem that plays a key role in immune regulation, metabolic health, and systemic inflammation. Nearly 70% of the body's immune cells are located within or near the intestinal tract, and the integrity of this gut–immune connection is vital for maintaining balance and longevity (97, 98). The makeup of the gut microbiota is influenced by genetics, diet, lifestyle, and environmental exposures, creating a unique microbial profile for each person (97). As age advances, the microbiome adjusts to the host's physiological changes; when this mutual adjustment stays balanced, it promotes healthy aging, but if disrupted, it can lead to chronic diseases (6). Aging usually involves a decrease in beneficial bacterial groups, like Firmicutes species, and an increase in proinflammatory, facultative anaerobes. These changes encourage the release of endotoxins such as lipopolysaccharide (LPS), which can cross a weakened intestinal barrier, fueling systemic low-grade inflammation (99). At the same time, microbial metabolism generates bioactive compounds that affect cardiovascular function. Higher levels of metabolites, such as trimethylamine N-oxide (TMAO), trimethyllysine, and phenylacetylglutamine (PAG), in the blood have been independently linked to a greater risk of major cardiovascular events (6). TMAO, formed either from microbial processing of dietary choline and L-carnitine into trimethylamine, followed by hepatic oxidation, or through direct absorption of preformed TMAO from certain foods such as fish and shellfish, has been confirmed in large clinical studies as a biomarker that predicts cardiovascular disease (100, 101). Diet plays a crucial role in shaping the microbiome and influencing these pathogenic pathways. Western dietary patterns, characterized by high consumption of red meat, processed foods, and saturated fats, promote TMAO-producing microbial communities, whereas Mediterranean and plant-based diets are associated with lower TMAO levels and a more diverse, anti-inflammatory microbial profile (102, 103). In a recent randomized crossover trial, DiMattia et al. demonstrated that the Mediterranean diet, even when containing moderate amounts of lean beef, increased gut microbiota diversity and lowered plasma and urinary TMAO levels compared with an average American diet (104). This suggests that the Mediterranean dietary pattern may attenuate TMAO generation by favorably modulating the gut microbiome. The Mediterranean diet has the longest and most extensive evidence base among the heart healthy patterns discussed here, spanning more than six decades of research. The first evidence linking the Mediterranean diet to reduced cardiovascular mortality emerged from the landmark Seven Countries Study in the late 1950s and early 1960s, with subsequent research in large Greek cohorts confirming a significant inverse association between Mediterranean diet adherence and all-cause mortality over long-term follow-up (105, 106). A recent systematic review and meta-analysis conducted for Italian national dietary guidelines has since confirmed that higher adherence to the Mediterranean diet is associated with lower all-cause mortality across the accumulated cohort evidence (107).

### Heart-healthy lifestyle

#### Physical activity

Physical activity is a fundamental pillar of healthy aging with clear, specific benefits for the cardiovascular system (108, 109). Epidemiological evidence from older populations indicates that regular physical activity is linked to reduced all-cause and cardiovascular mortality, with the most significant gains at moderate-to-intense activity levels, while further increases in intensity provide minimal additional mortality benefits (110). Recent syntheses similarly find that engaging in physical activity and having good cardiorespiratory fitness are reliably linked to lower mortality rates and increased lifespan (111). Physical activity favorably modifies multiple hallmarks of cardiovascular aging by lowering oxidative stress and reducing chronic low-grade inflammation, both of which are key factors in causing endothelial dysfunction, atherosclerosis, and arterial stiffening (112). Regular exercise also improves cardiometabolic health by enhancing insulin signaling and glucose metabolism in skeletal muscle, increasing insulin sensitivity (113). The ways physical activity promotes cardiovascular and overall health go beyond traditional risk factor management. Besides lowering hypertension, hyperglycemia, and dyslipidemia, exercise influences coagulation pathways to create a less thrombogenic environment, reduces inflammation and oxidative stress, and improves endothelial function (112, 114). Emerging evidence also indicates that physical activity may have anti-aging effects at the cellular level. Studies show a positive correlation between activity levels and telomere length, with athletes showing longer telomeres and higher telomerase activity compared to sedentary people (115, 116). Experimental data further indicate that short-term exercise can increase telomerase activity and decrease the expression of p16, a marker linked to cell cycle inhibition and senescence, as well as p53. This supports a cellular mechanism by which exercise might diminish senescence-related cellular changes (117).

#### Smoking cessation

Smoking remains one of the most powerful modifiable risk factors for cardiovascular disease, even in later life (118). In the long-lived population of Ikaria, current smoking prevalence is low (17% in men and 7% in women), while a substantial proportion of men report former smoking, suggesting that smoking cessation rather than lifelong abstinence may contribute meaningfully to cardiovascular health and aging (119). Evidence from large cohorts, such as the CHANCES consortium and the SMART study, shows that quitting smoking significantly lowers the risk of recurrent cardiovascular events and death compared to ongoing smoking (118, 120). Similarly, analyses from the Framingham Heart Study showed that former heavy smokers experience significant cardiovascular risk reduction within five years of cessation, though residual excess risk may persist compared with never-smokers (121). Mechanistically, smoking accelerates vascular aging through oxidative stress, endothelial dysfunction, and chronic inflammation, while cessation reverses many of these abnormalities—improving endothelial function, reducing oxidative injury, and restoring vascular elasticity (122).

#### Sleep hygiene

Sleep is a core pillar of health alongside diet and physical activity. Beyond circadian regulation, sleep influences vascular tone, autonomic balance, metabolic stability, and inflammatory signaling. Its importance is highlighted by its inclusion in “Life's Essential 8” (3). Epidemiologic evidence shows that seven to nine hours of sleep per night offer the lowest risk of adverse outcomes, with both shorter (<7 h) and longer (>8 h) sleep linked to higher all-cause mortality (123). Good sleep reduces the risk of cardiovascular disease (CVD) and major cardiac events, while insufficient or fragmented sleep—caused by deprivation, insomnia, or sleep-disordered breathing lessen nocturnal blood pressure dipping, shifts autonomic balance toward sympathetic dominance, increases hypothalamic–pituitary–adrenal activation, raises proinflammatory cytokines, disrupts glucose and lipid metabolism, and heightens oxidative stress (124, 125). These effects collectively promote endothelial dysfunction, arterial stiffness, and vascular aging (126, 127). Studies have shown that short sleepers face a 48% higher risk of coronary heart disease and insomnia symptoms raise CVD risk by up to 22%, while adherence to healthy sleep behaviors in a large U.S. population-based study by Li et al. was associated with significantly greater life expectancy, corresponding to approximately 4.7 additional years of life in men and 2.4 additional years in women compared with individuals with poor sleep patterns (125, 128, 129). Obstructive sleep apnea is common, affecting roughly 10 to 17% of middle aged and older medn and 3 to 9% of women, with prevalence rising alongside obesity (130). It highlights the cardiovascular toll of disordered sleep, with recurrent hypoxemia and arousals causing oxidative stress and sympathetic activation, leading to hypertension, arrhythmias, coronary heart disease and heart failure (131).

### Heart-healthy environment

#### Air pollution

Air pollution has become a major environmental factor influencing cardiovascular disease (CVD) (132). Inhaled pollutants—primarily fine particulate matter (PM2.5)—trigger a series of harmful biological responses, including oxidative stress, chronic inflammation, endothelial damage, and arterial stiffening (133, 134). These processes speed up cardiovascular aging and increase rates of illness and death (135). Both short-term and long-term exposure to polluted air are closely linked with cardiovascular events: prolonged PM2.5 exposure promotes subclinical atherosclerosis and rapid plaque growth, while brief pollution surges are connected to spikes in acute coronary syndromes and heart failure (136, 137). The impact of air pollution on life expectancy is substantial and quantifiable. Indirect calculations from the United States and the Netherlands estimate a loss of approximately 0.7 to 1.6 years per 10 µg/m^3^ increase in long-term exposure to PM2.5. Consistent with these estimates, longitudinal analyses of United States metropolitan areas show that a 10 µg/m^3^ reduction in PM2.5 over roughly two decades was associated with an increase in life expectancy of approximately 0.61 years, even after adjustment for socioeconomic and smoking-related factors (138). Mechanistically, pollution's negative effect on lifespan is mediated through ongoing inflammation, oxidative stress, and the worsening of conditions like diabetes and hypertension, all of which accelerate cardiovascular decline (133). At the cellular level, pollutants seem to affect key aging pathways. Exposure has been linked to telomere shortening and DNA methylation changes, both of which contribute to cellular aging and decreased regenerative ability (139, 140). These findings underscore the substantial impact that environmental quality has on shaping cardiovascular health and human lifespan.

#### Noise pollution

Noise pollution has become a major environmental factor influencing human health, second only to air pollution, with large-scale studies reliably associating prolonged noise exposure with increased risks of hypertension, diabetes, and atherosclerosis (141). Long-term exposure triggers biological pathways marked by oxidative stress and systemic inflammation, which speed up blood vessel damage and cardiovascular aging. Notably, the mechanisms behind noise-induced cardiovascular damage are similar to those of aging, involving oxidative stress, low-grade inflammation and metabolic issues (142). These combined processes promote CVD and share mechanistic overlap with biological aging pathways, though direct evidence linking noise exposure to reduced lifespan in humans is limited.

#### Climate

Climate change is increasingly recognized as a key factor driving cardiovascular aging. Temperature extremes—such as heat waves and cold spells—place significant stress on the cardiovascular system, especially in women and older adults (143). Heat stress causes dehydration, hemoconcentration, increased sympathetic activation, and higher cardiac workload, while cold exposure leads to vasoconstriction and increased blood pressure. Both extremes are linked to higher risk of myocardial infarction, arrhythmias, worsening heart failure, and cardiovascular death. Additionally, climate change worsens air quality with higher levels of particulate matter and ozone, which boost oxidative stress, systemic inflammation, endothelial dysfunction, and arterial stiffness—common pathways of vascular aging and inflammaging (144). Emerging concerns include finding microplastics in atheromatous plaques, indicating new pollution-related exposures that may speed up atherosclerosis and raise cardiovascular risk (145, 146). Climate-induced disruptions in food and water security also increase metabolic disorders such as hypertension and diabetes, further worsening age-related cardiovascular decline (144). Extreme weather events like floods, hurricanes, and wildfires also harm cardiovascular health by worsening air quality, increasing psychosocial stress, and disrupting access to medical care (147, 148). Importantly, the effects of climate change on cardiovascular health are not evenly distributed; socially and economically disadvantaged groups face the greatest dangers (144). Overall, these environmental and systemic stressors are mechanistically linked to accelerated biological aging and weakened cardiovascular resilience, with plausible but not yet directly demonstrated effects on lifespan, emphasizing the urgent need for combined strategies to reduce climate impacts and promote healthy aging.

#### Gamma (*γ*) radiation

High-dose gamma radiation is linked to increased cardiovascular disease and decreased longevity. Cohorts such as atomic-bomb survivors and patients treated with high-dose radiotherapy show long-term cardiac effects, including accelerated atherosclerosis, higher rates of myocardial infarction, cardiomyopathy, malignancy and heart failure. The biological mechanisms underlying these effects include radiation-induced damage to the endothelium, inflammation, accelerated plaque formation, and ongoing impairment of myocardial and vascular repair processes (149). In contrast, the health impacts of low-dose or chronic low-rate gamma exposure remain debated. The widely accepted linear no-threshold (LNT) model suggests that any small dose carries some risk. Still, a substantial amount of experimental research reports adaptive responses at low exposure, consistent with hormesis—a dose response phenomenon in which low level stressors elicit beneficial cellular adaptations, while higher exposures become harmful (150, 151). Proposed mechanisms for potential low-dose advantages include temporary stimulation of DNA-repair pathways, increased antioxidant defenses, improved immune surveillance, and reductions in certain chromosomal damage markers after a priming low dose (151). Animal studies have sometimes shown modest increases in lifespan or decreases in mutation rates after very low, continuous irradiation, and some ecological data have associated higher-background regions with different disease or mortality patterns (152). However, several epidemiologic meta-analyses of human cohorts indicate a small but real increase in cardiovascular and other health risks even at low doses and dose rates (153, 154). In medical practice, the controlled and targeted application of ionizing radiation can be beneficial for certain cardiac conditions, like intracoronary radiation to prevent restenosis after angioplasty and modern stereotactic radiotherapy protocols tested for refractory cardiac arrhythmias (155). Localized population studies have shown links between higher measured background gamma levels and greater longevity or different disease rates, and older regional comparisons have observed that higher-radiation areas, such as the Rocky Mountain states, had lower cancer mortality compared with lower background exposure, such as the Gulf coast (156, 157). These findings are hypothesis-generating but limited by ecological confounding and cannot establish direct causality. Overall, the evidence for the net benefits of low-dose gamma exposure in humans remains unresolved. Further rigorous, well-controlled studies are needed to clarify these discrepancies.

### Current challenges

Understanding the mechanisms that control cardiovascular aging and longevity remains an ongoing challenge due to genetic complexity, environmental diversity, and biological variability. Although genes such as APOE and FOXO3 are consistently associated with longer lifespan, the search for more “longevity genes” is slowed by the polygenic nature of aging. GWAS has often yielded inconsistent results across different populations because of variations in ancestry, environmental exposures, and phenotype definitions, which makes replication and establishing causality difficult (158). Additionally, longevity is not solely explained by inherited differences—epigenetic changes, telomere shortening, and somatic mutations like clonal hematopoiesis develop with age, blurring the line between genetic predisposition and acquired molecular damage (159, 160). Pathways like mTOR, sirtuins, and insulin/IGF-1 signaling remain among the most promising molecular targets for promoting longevity, but their modulation involves significant biological trade-offs. While inhibiting mTOR or activating sirtuins has extended lifespan in model organisms, these same interventions can disrupt metabolic balance, weaken immune function, or alter cellular repair mechanisms in humans, highlighting the challenge of translating experimental success into safe clinical use (161). Similarly, biomarkers of biological aging such as telomere length and epigenetic clocks, discussed above, remain limited by variability across tissues, methodological inconsistencies, and uncertain prognostic value in clinical settings (162). Adding to these scientific hurdles is the persistent lack of diversity in genetic studies, which are predominantly based on cohorts of European ancestry (163). This imbalance restricts understanding of how ancestry-specific and sex-related genetic and hormonal factors influence cardiovascular aging and longevity. Further complicating these biological uncertainties are environmental stressors—such as air pollution, temperature extremes, and chronic noise exposure that accelerate vascular aging through oxidative stress and inflammation. However, separating their direct biological effects from overlapping socioeconomic and behavioral factors remains a persistent challenge.

### Future direction

Future research in cardiovascular longevity will depend on multidisciplinary integration, utilizing artificial intelligence (AI) and advanced computational tools to analyze complex biological and environmental data. AI-driven models, especially deep learning and network-based analytics, can identify nonlinear interactions among genes, metabolites, and environmental factors that are not detectable with traditional statistical methods (164). These methods will help discover new longevity-related genes and pathways, providing mechanistic insights into how genomic differences influence cardiovascular resilience. Combining AI with single-cell sequencing, proteomics, and spatial transcriptomics will enable detailed mapping of cellular aging across tissues with unparalleled accuracy. Machine learning–based predictive models could improve epigenetic and transcriptomic aging clocks, turning them into clinically useful biomarkers that accurately reflect biological cardiovascular age (165). Integrating this data with digital health tools like wearable sensors and continuous physiological monitoring will create real-time, personalized risk profiles and intervention strategies (166). In the future, AI-driven drug discovery and precision gene editing methods, including CRISPR targeting of pathways such as FOXO3, SIRT1, and mTOR, hold promise for developing targeted therapies to slow vascular aging and support healthy longevity.

## Conclusion

In conclusion, cardiovascular aging and lifespan are shaped by an interconnected mix of genetic, mechanistic, and modifiable factors. Grasping the distinct roles of these components is essential for understanding their relationship. Genetic factors such as APOE and FOXO3, along with shared biological processes like oxidative stress, inflammaging, metabolic issues, vascular changes, and genetic/epigenetic control, form the biological base on which lifestyle and environmental influences act. This review emphasizes evidence supporting cardiovascular aging separately from the less clear evidence linking specific factors directly to longevity. This distinction, often unclear in current research, is key to identifying interventions that might extend lifespan versus those solely improving cardiovascular health. Modifiable factors include heart-healthy diets, regular exercise, quitting smoking, good sleep, and managing environmental exposures. These are practical, evidence-supported targets consistently linked to lower cardiovascular mortality and sometimes to indicators of healthy aging or longer life. Although genetic and epigenetic factors are mostly non-modifiable now, future progress may allow personalized risk assessments. Significant uncertainties remain, especially in translating molecular longevity pathways into safe clinical treatments and in applying findings mainly from European-ancestry groups. Ultimately, advancing cardiovascular health and longevity depends on integrating genetic insights with ongoing lifestyle and environmental changes, guided by evidence specific to the targeted outcome—cardiovascular aging or longevity.
